# Supplementary material for: Evolutionary remodelling of N‐terminal domain loops fine‐tunes SARS‐CoV‐2 spike
Source: EMBO Rep. 2022 Sep 1;23(10):e54322. doi: 10.15252/embr.202154322 (PMC9535765; doi:10.15252/embr.202154322)
Supplement: Supplementary file 1 — Expanded View Figures PDF [file EMBR-23-0-s002.pdf]

Expanded View Figures

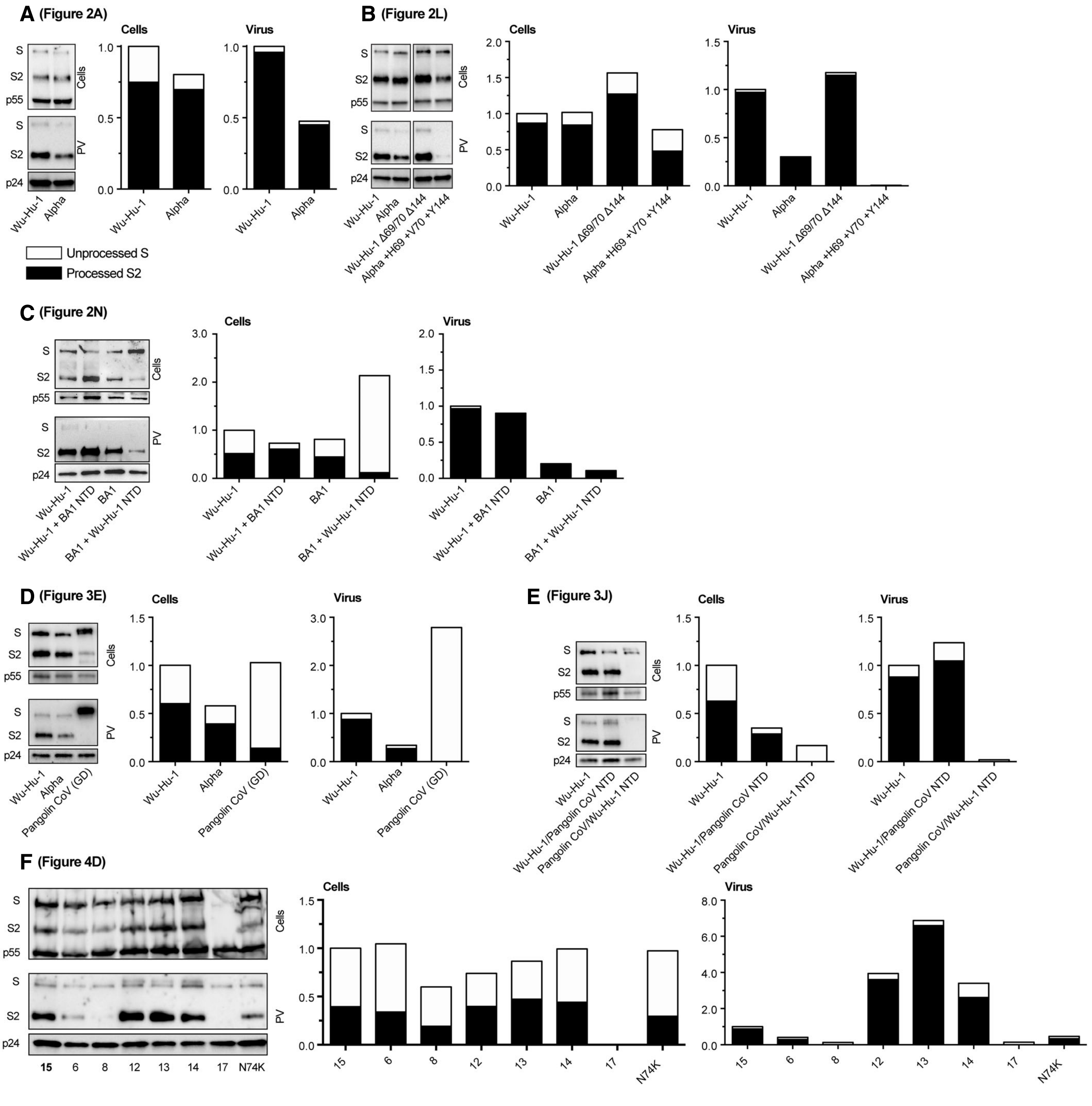

Figure EV1.

◀ **Figure EV1. Quantification of spike expression and incorporation into pseudovirus.**

Quantification of Western blots from main text figures, blot images are reproduced alongside their respective quantification.

A Quantification of Fig 2A.
B Quantification of Fig 2L.
C Quantification of Fig 2N.
D Quantification of Fig 3E.
E Quantification of Fig 3J.
F Quantification of Fig 4D.

Data information: In each case sample loading is corrected using p55 (cell samples) or p24 (PV pellets) band intensities. Total spike (sum band intensities of unprocessed and processed spike) normalised to Wu-Hu-1 control. In each bar, the portion coloured white represents the relative quantity of unprocessed spike (S), whereas the black portion represents S1/S2 proteolytically cleaved spike (S2).

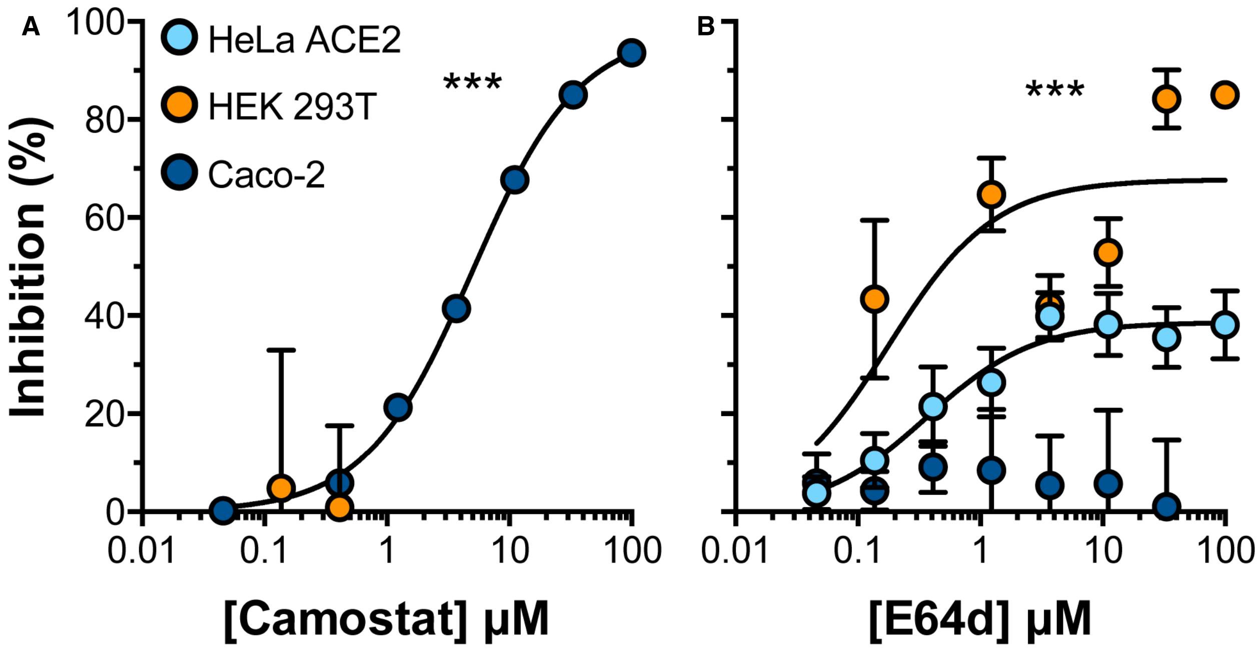

Figure EV2. SARS-CoV-2 entry into Caco-2 cells is TMPRSS2 dependent.

PV bearing Wu-Hu-1 spike protein were used to infect the stated cell lines and were treated with a serial dilution of either (A) Camostat mesylate (inhibitor of TMPRSS2 dependent entry at the cell surface) or (B) E64d (inhibitor of cathepsin-dependent entry in the endosome). Data are expressed as mean per cent inhibition relative to vehicle only control, $n = 3$ biological repeats. Error bars indicate standard error of the mean. Curve fitting performed in GraphPad Prism. Curves determined to be statistically significant by F-test, $^{***}P \leq 0.001$.

 © 2022 The Authors

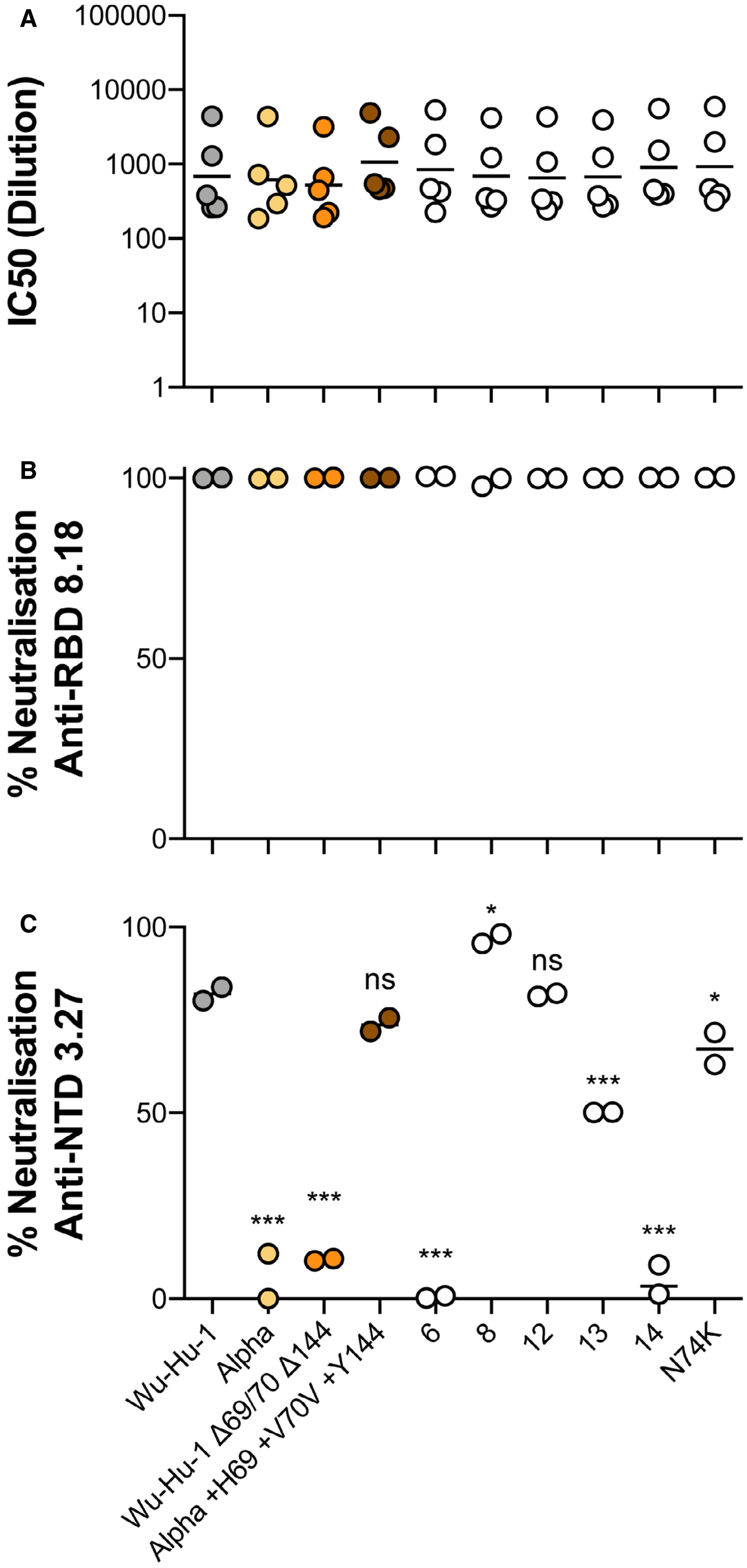

Figure EV3. Anti-spike neutralisation of N2 Loop variants.

A PVs bearing Wu-Hu-1, Alpha, Wu-Hu-1 Δ69/70 Δ144, Alpha +H69 +V70 +Y144, and the N2 loop variants were subjected to neutralisation by a set of five SARS-CoV-2 convalescent sera. Plots display individual IC50 values calculated from a single serial dilution neutralisation assay. Volumes of sera did not permit a repeat experiment.

B, C Single concentration neutralisation by 2 μg/ml anti-RBD mAb 8.18 (B) and 17 μg/ml anti-NTD mAb 3.27 (C).

Data information: Data points represent mean neutralisation from $n = 2$ biological repeats. Statistical analysis (ANOVA, compared to Wu-Hu-1) performed in GraphPad Prism.*$P \leq 0.05$, **$P \leq 0.01$, ***$P \leq 0.001$; ns, not significant.

© 2022 The Authors
