# Supplementary material for: Evolutionary remodelling of N‐terminal domain loops fine‐tunes SARS‐CoV‐2 spike
Source: EMBO Rep. 2022 Sep 1;23(10):e54322. doi: 10.15252/embr.202154322 (PMC9535765; doi:10.15252/embr.202154322)
Supplement: Supplementary file 5 — Source Data for Figure 2 [file EMBR-23-0-s007.pdf]

Raw western blot data: Figure 2

Boxes denote crop used in main figure.

Protein ladder = NEB Prestained Broad Range Protein Standard (10-250kDa)

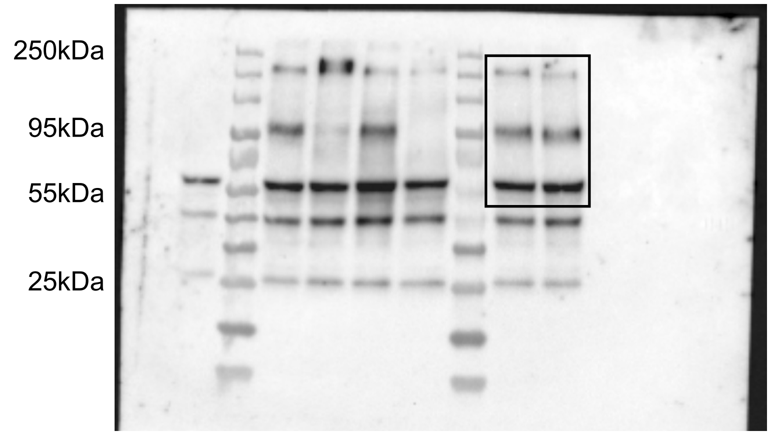

Figure 2A
Cell lysates, Spike and p55

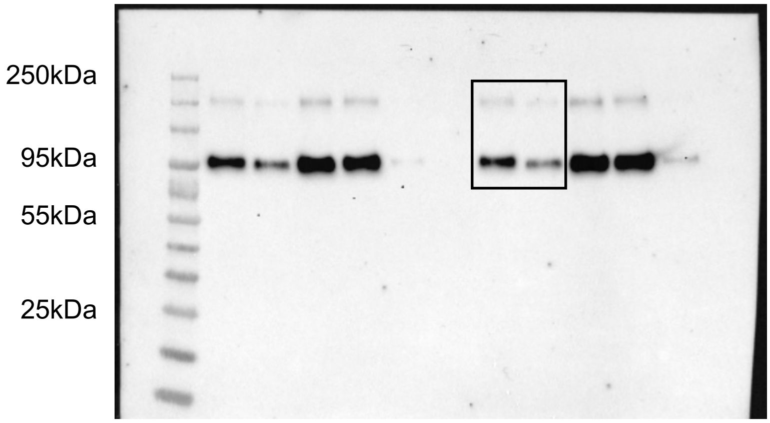

Figure 2A
PV, Spike

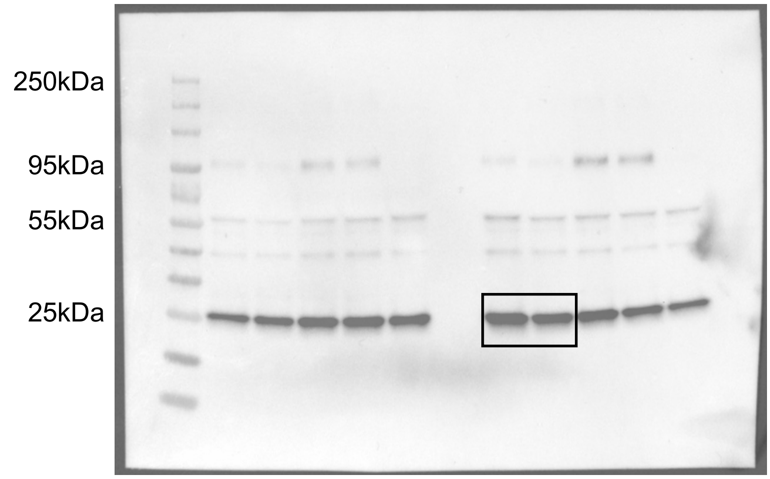

Figure 2A
PV, p24

Raw western blot data: Figure 2

Boxes denote crop used in main figure.

Protein ladder = NEB Prestained Broad Range Protein Standard (10-250kDa)

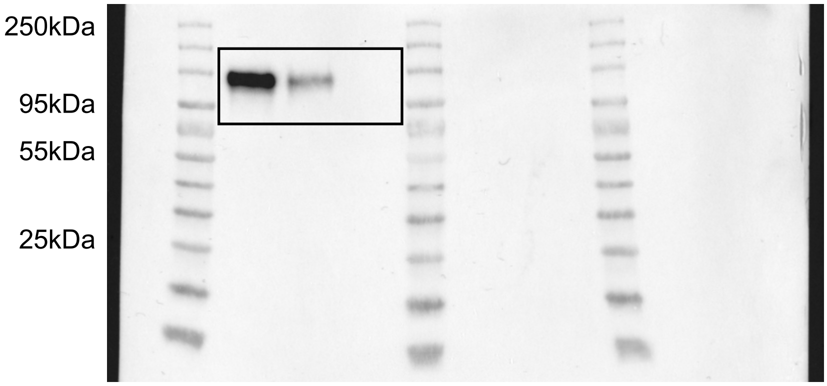

Figure 2F
ACE2

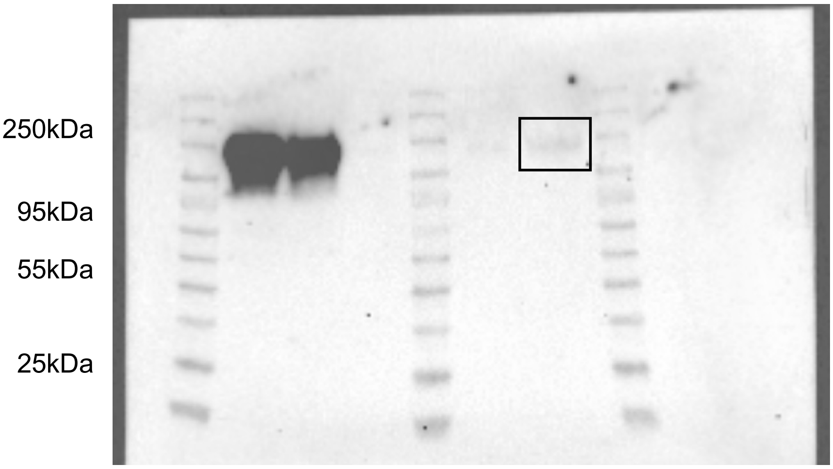

Figure 2F
ACE2 long exposure

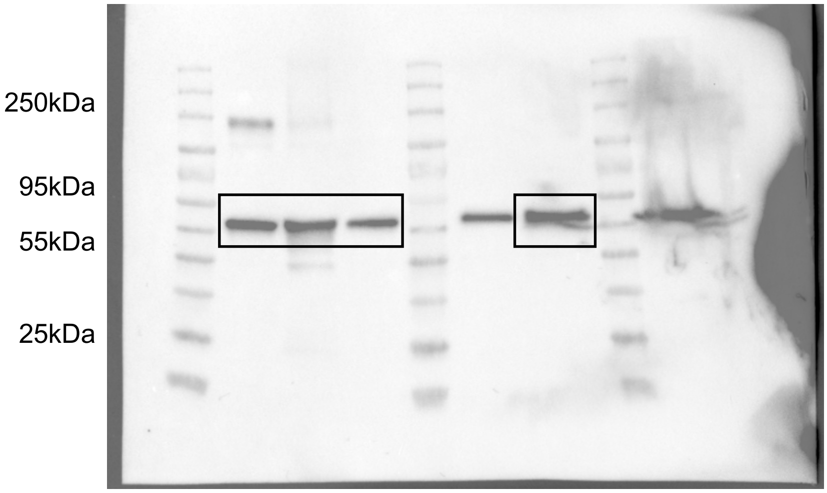

Figure 2F
Actin

Raw western blot data: Figure 2

Boxes denote crop used in main figure.

Protein ladder = NEB Prestained Broad Range Protein Standard (10-250kDa)

250kDa
95kDa
55kDa
25kDa

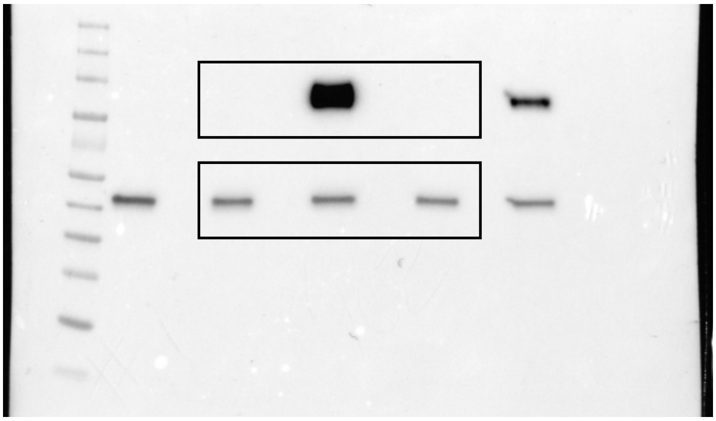

Figure 2J
ACE2 and Actin

250kDa
95kDa
55kDa
25kDa

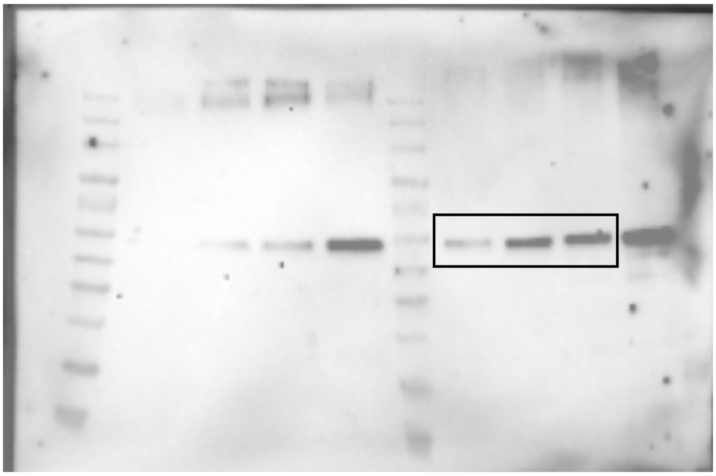

Figure 2F
TMPRSS2

250kDa
95kDa
55kDa
25kDa

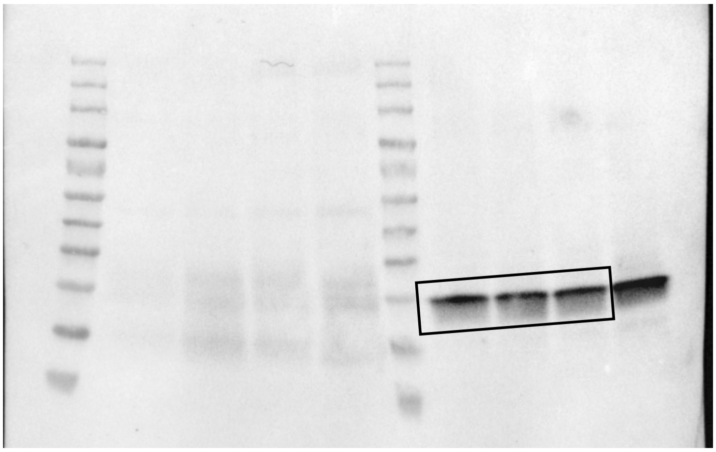

Figure 2F
CD81

Raw western blot data: Figure 2

Boxes denote crop used in main figure.

Protein ladder = NEB Prestained Broad Range Protein Standard (10-250kDa)

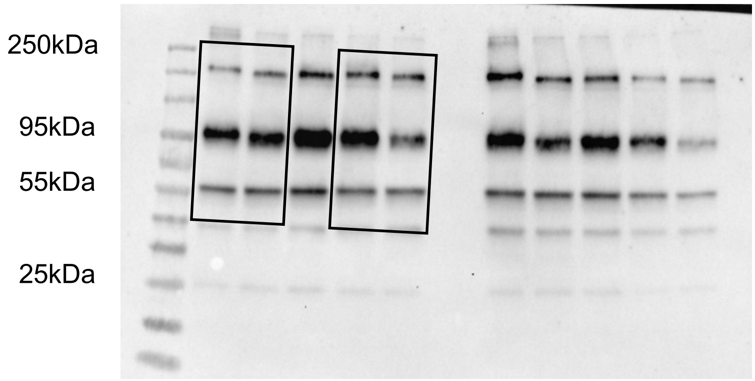

Figure 2L
Cell lysates, Spike and p55

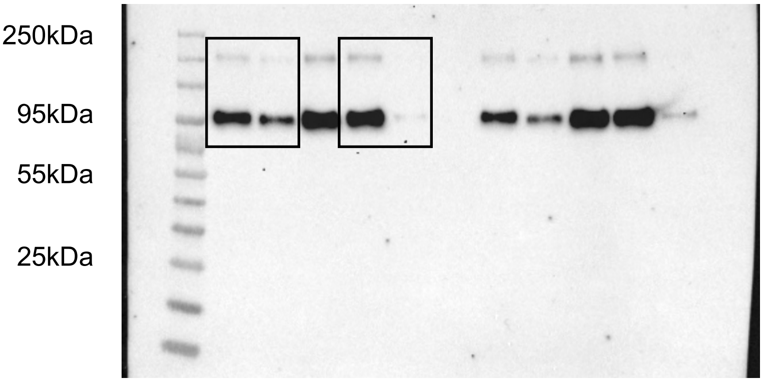

Figure 2F
PV, Spike

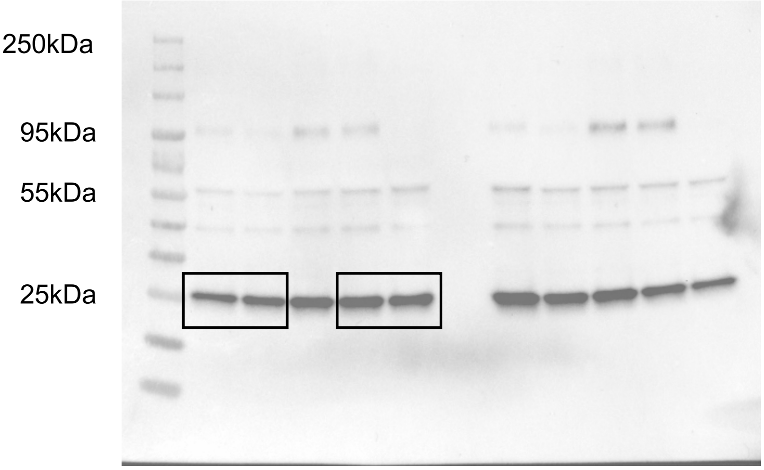

Figure 2F
PV, p24

Raw western blot data: Figure 2
Boxes denote crop used in main figure.
Protein ladder = BIORAD Precision Plus Protein Dual Xtra

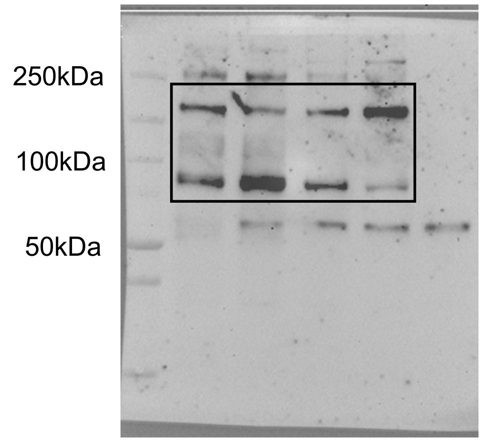

Figure 2N
Cell lysates, Spike

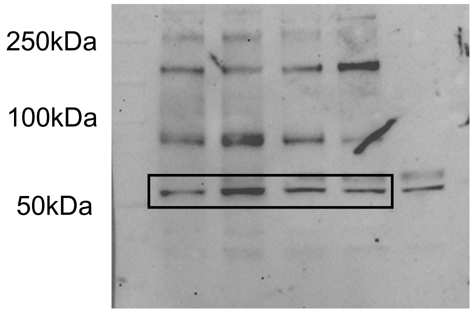

Figure 2N
Cell lysates, p55

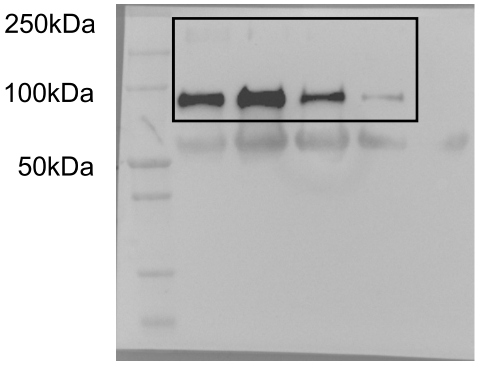

Figure 2N
PV, Spike

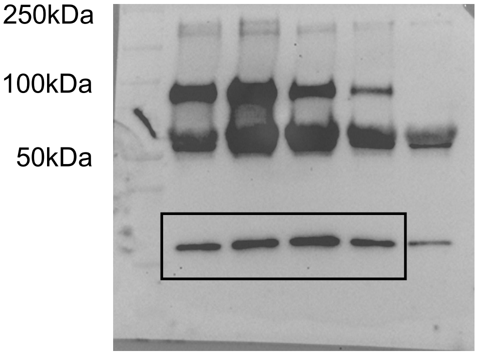

Figure 2N
PV, p24
