# Supplementary material for: Evolutionary remodelling of N‐terminal domain loops fine‐tunes SARS‐CoV‐2 spike
Source: EMBO Rep. 2022 Sep 1;23(10):e54322. doi: 10.15252/embr.202154322 (PMC9535765; doi:10.15252/embr.202154322)
Supplement: Supplementary file 6 — Source Data for Figure 3 [file EMBR-23-0-s001.pdf]

Raw western blot data: Figure 3

Boxes denote crop used in main figure.

Protein ladder = NEB Prestained Broad Range Protein Standard (10-250kDa)

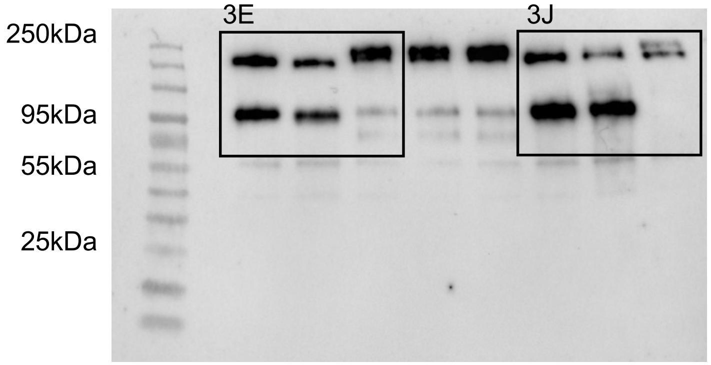

Figure 3E and 3J
Cell lysates, Spike

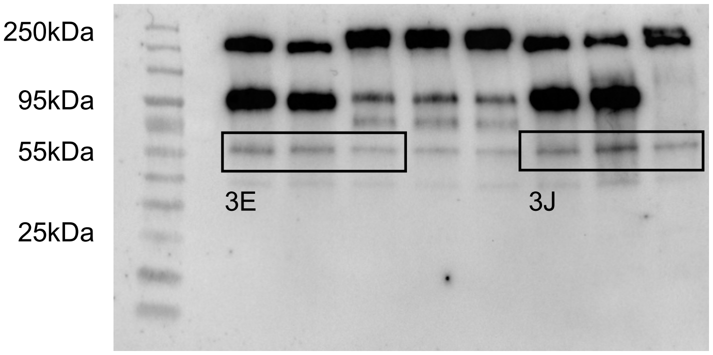

Figure 3E and 3J
Cell lysates, p55

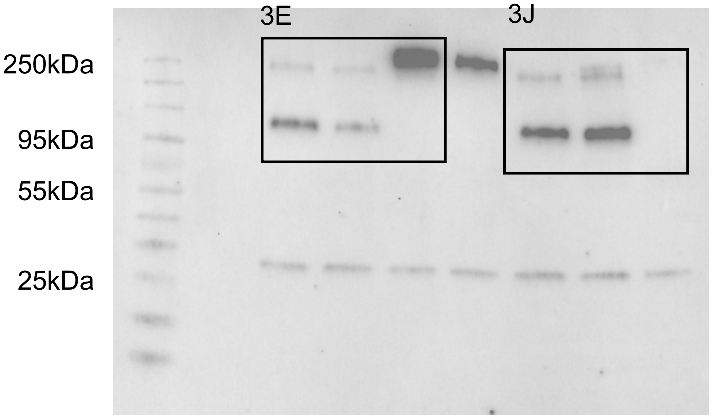

Figure 3E and 3J
PV, Spike

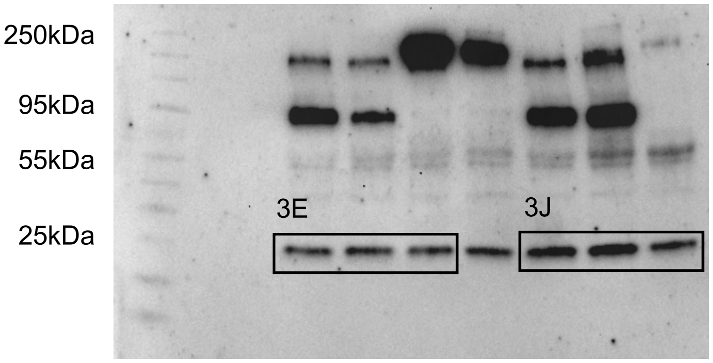

Figure 3E and 3J
PV, p24
