# Supplementary material for: Evolutionary remodelling of N‐terminal domain loops fine‐tunes SARS‐CoV‐2 spike
Source: EMBO Rep. 2022 Sep 1;23(10):e54322. doi: 10.15252/embr.202154322 (PMC9535765; doi:10.15252/embr.202154322)
Supplement: Supplementary file 7 — Source Data for Figure 4 [file EMBR-23-0-s006.pdf]

Raw western blot data: Figure 4
Boxes denote crop used in main figure.
Protein ladder = NEB Prestained Broad Range Protein Standard (10-250kDa)

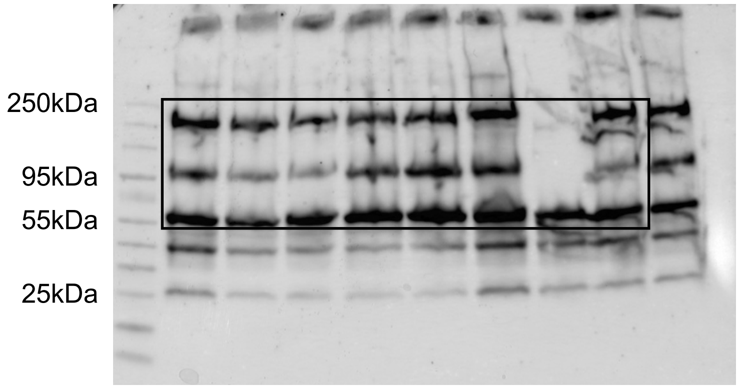

Figure 4D
Cell lysates, Spike and p55

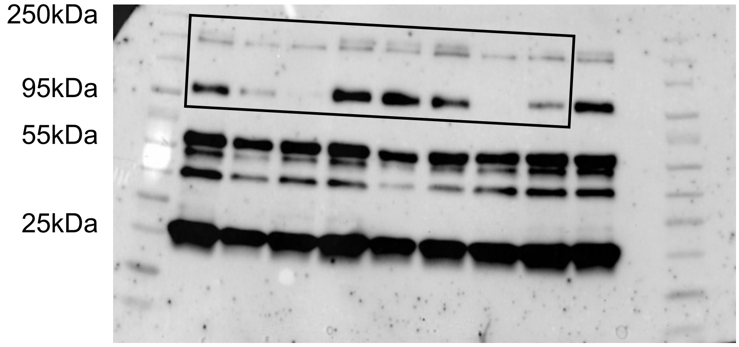

Figure 4D
PV, Spike

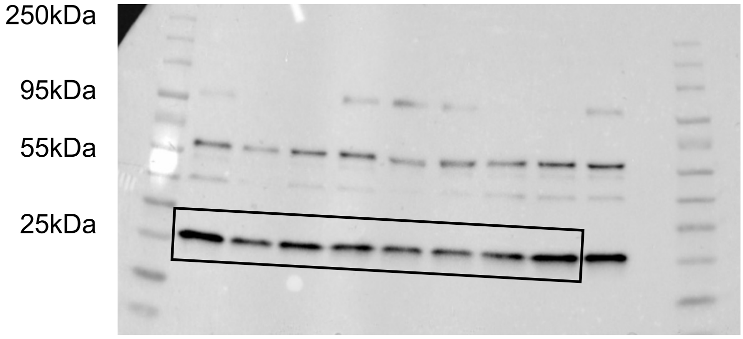

Figure 4D
PV, p24
